# CpG site- and methylation level-dependent measurement variability across three bisulfite-based DNA methylation assays

**DOI:** 10.1080/15592294.2026.2753261

**Published:** 2026-10-29

**Authors:** Fatemeh Rangani, Maria Needhamsen, Lara Kular, Maja Jagodic, Majid Pahlevan Kakhki

**Affiliations:** Department of Clinical Neuroscience, Karolinska Institutet, and Center for Molecular Medicine, Karolinska University Hospital, Stockholm, Sweden

**Keywords:** DNA methylation, CpG, epigenetics, pyrosequencing, Illumina EPIC array, QIAseq Targeted Methyl Panel

## Abstract

Accurate quantification of DNA methylation at specific CpG sites is essential for epigenetic studies, yet measurements can vary depending on assay design and experimental context. Here, we examined locus-specific DNA methylation quantification across three widely used bisulfite-based assays, Illumina Infinium MethylationEPIC array, QIAseq targeted methyl sequencing (TMS), and pyrosequencing, using identical DNA samples. This controlled framework enabled evaluation of method-dependent measurement behavior across CpG sites and a range of methylation levels while minimizing biological variability. Across shared CpGs, all three platforms consistently captured relative methylation trends; however, absolute methylation values differed systematically in a CpG- and methylation-dependent manner. Pyrosequencing generally reported higher methylation levels compared with TMS, whereas EPIC array measurements showed increased variability at low methylation levels (<10%), resulting in reduced inter-method agreement in this range. In contrast, concordance between methods improved at moderate-to-high methylation levels. Error estimates and agreement analyses further indicated that measurement variability was not uniform across CpGs, highlighting site-specific and methylation-level-dependent effects. Together, these results demonstrate that locus-specific DNA methylation quantification using bisulfite-based assays is influenced by local CpG context and methylation level. While relative methylation patterns are preserved across platforms, absolute values are not directly interchangeable, particularly at low methylation levels. These findings underscore the importance of context-aware interpretation and orthogonal validation when precise, locus-specific DNA methylation quantification is required.

## Introduction

DNA methylation, the covalent addition of a methyl group to cytosine residues within CpG dinucleotides, is a fundamental epigenetic modification involved in the regulation of gene expression, chromatin structure, and genome stability [1]. Aberrant methylation patterns are implicated in diverse human diseases, including cancer, neurological disorders, and autoimmune conditions [2–5] and are also influenced by environmental exposures and lifestyle factors. Consequently, precise and scalable methods for DNA methylation manipulation and quantification are essential for both basic research and translational applications. Locus-specific epigenome editing using CRISPR/dCas9-based DNA methylation modifiers allows the establishment of controlled methylation states at the same locus, thereby providing an experimental system to examine method-dependent variability in DNA methylation measurements across a defined methylation spectrum while minimizing biological variability. Importantly, the performance of DNA methylation measurement methods can vary depending on methylation levels, posing challenges not only for detecting subtle natural differences, typically observed in EWAS of non-cancer diseases, but also when methylation is experimentally manipulated [6,7].

Over the years, several methods have been developed for accurate and quantitative assessment of DNA methylation [8]. These methods evolved through four main approaches: affinity enrichment, endonuclease digestion, bisulfite-dependent, and bisulfite-free methods [8]. Affinity enrichment (e.g., MeDIP-seq [9], MBD-seq [10]) captures methylated DNA via antibodies or binding domains, while endonuclease digestion (e.g., MSRE [11], SCRAM [12]) distinguishes methylated regions using methylation-sensitive enzymes. Bisulfite-dependent methods (e.g., RRBS [13], WGBS [14], pyrosequencing [15], targeted methyl sequencing, and the EPIC array) convert unmethylated cytosines to uracil for single-base or high-resolution mapping. Enzyme-based bisulfite-free methods (e.g., TAPS [16] and EM-seq [17]) offer improved accuracy and reduced DNA degradation. Although many widely used DNA methylation assays rely on bisulfite conversion, they differ substantially in assay design, detection chemistry, throughput, and sources of technical biases, and are frequently employed in complementary roles across discovery and validation phases [18,19].

Bisulfite pyrosequencing is a well-established method for quantifying DNA methylation at single-CpG resolution, with reliable quantitative performance and low technical variability at predefined regions. Its utility as a targeted validation tool is well recognized, particularly for confirming relative methylation differences detected through genome-scale approaches. Nevertheless, its limited multiplexing capacity, short amplicon design requirements, and dependence on custom assay development restrict its applicability in large-scale or high-throughput settings [15]. The EPIC array, in contrast, provides a cost effective and high-throughput solution for genome-wide methylation profiling across more than 850,000 CpG sites [20]. Its fixed-probe architecture, however, is inherently biased toward promoter regions and CpG islands, with limited coverage of enhancers, gene bodies, or intergenic regulatory elements [21]. Moreover, because array probes interrogate only a subset of CpGs per region, quantitative interpretation of methylation levels can be more uncertain in low-CpG-density or partially methylated regions, where local heterogeneity is not fully captured. TMS methods, such as the QIAseq Targeted Methyl Panel, offer a flexible intermediate between genome-wide discovery and site-specific validation. This method applies bisulfite conversion followed by targeted amplification and next-generation sequencing to interrogate user-defined regions at single-nucleotide resolution [22]. Advantages of TMS include customizable locus selection, relatively high throughput, high sequencing depth compared with genome-wide sequencing approaches, and the incorporation of unique molecular identifiers (UMIs), which improve quantification accuracy by correcting for PCR duplicates. Nonetheless, bisulfite-induced DNA degradation, variability in capture efficiency, and the complexity of enrichment workflows remain challenges that can introduce locus-specific bias.

Although prior studies have reported general concordance among widely used DNA methylation methods [18,19], particularly at well-characterized CpG sites, differences in absolute methylation values, dynamic range, and site-specific sensitivity remain common. Systematic comparison under controlled and standardized conditions is essential for meaningful method evaluation and informed method selection. Such assessments are particularly valuable for evaluating each method’s ability to detect fine-scale, incremental changes in DNA methylation, including those associated with complex traits or introduced by epigenome-editing tools. Ultimately, comparative performance data can help guide methodological choices in epigenetic studies, whether the aim is high-throughput screening, focused discovery, or precise validation of site-specific methylation changes. In this study, we perform a controlled, locus-specific comparison to assess how CpG site characteristics and methylation level influence inter-method variability in DNA methylation quantification.

## Materials and methods

### Sample preparation and DNA extraction

The samples and experimental workflow used in this study were part of a previously published investigation on CRISPR/dCas9-mediated epigenome editing (Suppl. Table 1) [23]. Briefly, HEK293T cells were transfected with different CRISPR/dCas9-based constructs and gRNAs. GFP+ or BFP+ cells were sorted by flow cytometry on day 3 post-transfection, replated, and subsequently harvested on days 7, 14, 21, and 30 for further analysis. DNA was extracted using the AllPrep DNA/RNA/Protein Mini Kit (QIAGEN) following the manufacturer’s instructions, and its quantity and quality were assessed using a Qubit Fluorimeter (Life Technologies). The details of the CpG sites covered by each method are provided in Supplementary Table 2. The same DNA samples were used for all three DNA methylation measurement methods.

## Pyrosequencing

To evaluate the DNA methylation at the targeted *BACH2* promoter, genomic DNA (200 ng, *n* = 90) was subjected to bisulfite conversion using the EZ DNA Methylation-Gold Kit (Zymo Research), according to the manufacturer’s protocol. Subsequently, ~10 ng of bisulfite-converted DNA was amplified via PCR using the PyroMark PCR Kit (QIAGEN), with 5 pmol each of the forward and 5′-biotinylated reverse primer, under the following cycling conditions: initial denaturation at 95°C for 15 min; 50 cycles of 94°C for 30 s, 48°C for 30 s, and 72°C for 30 s; and a final extension at 72°C for 10 min, with primers targeting a CpG-rich region within the *BACH2* promoter encompassing 13 CpG sites. The forward primer sequence was 5′-TTATTGTGAATGGGGA-3′, and the biotinylated reverse primer sequence was 5′-ACTACTACTACTAAAAC-3′. Pyrosequencing primers and probes were designed using PyroMark Design software (QIAGEN). Pyrosequencing was performed on the PyroMark Q96 Advanced system using PyroMark Gold 96 reagents (QIAGEN) with the sequencing primer 5′-GATTGAGAGTGTGGG-3′. Methylation levels at individual CpG sites were quantified using PyroMark software. Pyrosequencing methylation values were normalized per plate against included 0%, 50%, and 100% methylation standards. For each plate, a linear standard curve (signal vs. known methylation percentage) was fit and used to convert raw signals to methylation percentages. Full-precision pyrosequencing values are provided in Supplementary Tables 3 & 4.

## QIAseq targeted methyl panel (TMS)

High-resolution, targeted DNA methylation profiling was performed using the QIAseq Targeted Methyl Panel (QIAGEN), hereafter referred to as TMS. The panel was designed to cover a 585 bp region of the promoter of *BACH2* gene on chromosome 6 (hg38: chr6:90,296,566–90,297,151), which includes 86 CpG sites. The design included four primer pairs, with two on the plus strand and two on the minus strand. A total of 110 ng of DNA (*n* = 90) was bisulfite-converted using the EpiTect Fast Bisulfite Conversion Kit (QIAGEN). Notably, different bisulfite conversion kits were used across platforms (EpiTect Fast for TMS; EZ DNA Methylation-Gold for pyrosequencing and EPIC array), due to platform-specific protocol requirements and manufacturer recommendations, which precluded full standardization across assays. Libraries were constructed with UMI-labeled fragments and amplified for 18 PCR cycles. Library quality was assessed using an Agilent 2100 Bioanalyzer (Agilent Technologies) and quantified using a KAPA Biosystems next-generation sequencing library qPCR kit. Libraries were pooled in equimolar concentrations, and a 10 pM pooled library was sequenced on the Illumina NovaSeq 6000 platform with 150 bp paired-end reads. Raw reads were analyzed using the QIAGEN CLC Genomics Workbench, and CpG sites with less than 10× coverage were excluded.

## EPIC array

Genome-wide DNA methylation was assessed using the Infinium MethylationEPIC v1.0 BeadChip (*n* = 33) at NGI (Uppsala, Sweden). For EPIC array analysis, 500 ng of genomic DNA per sample was bisulfite-converted using the EZ DNA Methylation-Gold Kit (Zymo Research) according to the manufacturer’s instructions. The 33 samples analyzed on the EPIC array represent a subset of the same 90 samples included in the TMS and pyrosequencing analyses. In the *BACH2* locus, eight CpG probes mapping to the genomic region were covered on the EPIC array (Suppl. Table 2). Raw data were normalized and analyzed using the default Illumina normalization procedure implemented in the Bioconductor minfi package in R. All analyses were performed using R (v4.3.1) with standard scientific libraries.

## Statistical analysis

All statistical analyses were conducted in R (v4.3.1). Continuous variables are presented as mean ± standard deviation (SD) unless otherwise stated. Normality of distributions was assessed using the Shapiro–Wilk test, and non-parametric tests were applied when normality assumptions were not met. For paired comparisons of methylation values between methods, the Wilcoxon signed-rank test was employed, while distributional differences were evaluated using the Kolmogorov–Smirnov test. Homogeneity of variance was assessed with Levene’s test, and heteroscedasticity was evaluated using the Breusch–Pagan test. Inter-method agreement was quantified using Bland–Altman analysis, reporting mean difference and 95% limits of agreement. Correlation analyses included Pearson’s r to assess linear associations and Spearman’s ρ to evaluate rank-based concordance. Platform-wide method reliability was assessed using the Friedman test for repeated measures, with Kendall’s W reported as an effect size. Post-hoc pairwise comparisons were adjusted using Holm’s correction. Intraclass Correlation Coefficient (ICC) was calculated using a two-way mixed-effects model for both absolute agreement [ICC(A,1)] and consistency [ICC(C,1)], and 95% confidence intervals were reported. Inter-method variability at individual CpG sites was further quantified using error metrics, including Mean Absolute Error (MAE) and Root Mean Square Error (RMSE). For data visualization, principal component analysis (PCA) was performed on z-score‑scaled methylation values using *prcomp()*, and clustering of high-methylation samples was highlighted with convex hulls. Statistical significance was defined as *p* < 0.05 unless otherwise indicated.

## Results

### Designed strategy for cross-methodology comparison of DNA methylation

To assess inter-platform variability in DNA methylation quantification, we compared three widely used bisulfite-based assays, pyrosequencing, Illumina Infinium Methylation EPIC array, and QIAseq targeted methyl sequencing (TMS), using identical DNA samples generated by CRISPR/dCas9-mediated epigenome editing at the *BACH2* promoter in HEK293T cells (Figure 1(a)). This controlled framework enables locus-specific evaluation of method-dependent measurement behavior across a range of methylation levels while minimizing biological variability. Targeted DNA methylation editing was performed using *BACH2*-specific guide RNA to generate localized methylation changes, producing samples spanning low to high methylation states as previously described [23]. Genomic DNA from the same samples was analyzed across all platforms to ensure direct comparability (Figure 1(a,b), Suppl. Table 1). Although multiple CpG sites within the *BACH2* promoter were interrogated, coverage differed across platforms due to assay-specific design constraints (Figure 1(c), Suppl. Table 2). Thirteen CpGs were shared between TMS and pyrosequencing, eight between TMS and EPIC array, and two across all three platforms, limiting three-way comparisons to these overlapping sites. This design enables systematic evaluation of inter-platform variability at shared CpGs within a defined regulatory region, providing a framework to assess the influence of CpG context and methylation level on measurement concordance. Consistent with the CRISPR/dCas9-edited methylation states generated at this locus, the sampled CpGs were predominantly low-methylated: 76.0% of all TMS measurements and 79.1% of profiled CpGs (68/86) had methylation ≤ 10% (Suppl. Figure 1). As higher-methylation CpGs were comparatively underrepresented in this dataset, findings for that range are based on fewer measurements (Suppl. Tables 3 & 4, Suppl. Figure 1).

**Figure 1.**
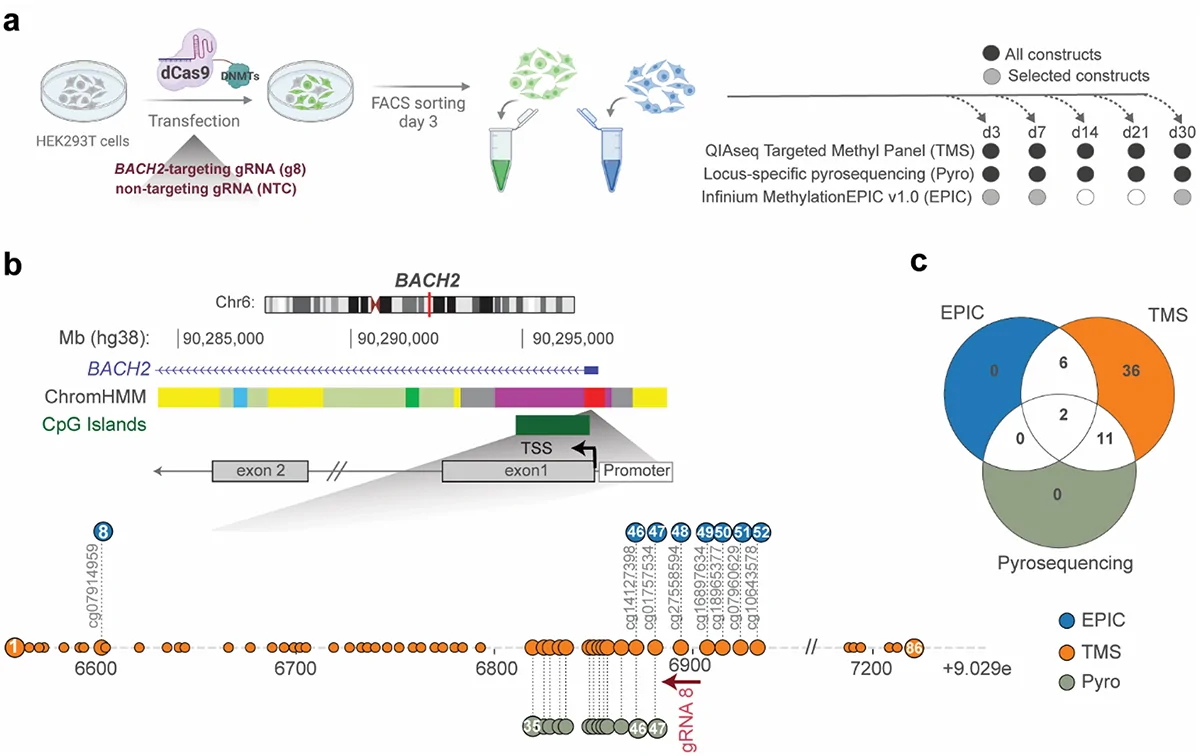
(**a**) Schematic representation of the designed experiment. (**b**) Genomic localization of *BACH2* promoter CpGs targeted by the specific *BACH2*-targeting gRNA 8 (g8). The illustration showing the location and the map of the CpG sites measured by EPIC array, TMS, and pyrosequencing. Genomic features include promoter, exon 1, CpG islands, and ChromHMM-annotated regulatory elements. (**c**) Venn diagram displaying the overlap of CpG sites covered by each method.

## Pyrosequencing reports higher methylation values but remains strongly correlated with TMS

To characterize differences between TMS and pyrosequencing in locus‑specific DNA methylation quantification, we performed a comparative analysis across 13 CpG sites shared between both methods in 90 samples spanning a range of methylation levels at the *BACH2* promoter. Read depth across the 86 CpG sites and 90 samples was high overall (median 1,720×; mean 3,165×; range 18–43,096×), with 37-fold variation in median depth across sites (Suppl. Figure 1). Full-precision TMS values are provided in Supplementary Tables 3 & 4. Global methylation density plots revealed a systematic shift in absolute values, with pyrosequencing consistently reporting higher methylation levels (mean = 17.5%) compared with TMS (mean = 10.7%) across samples (Figure 2(a)). Levene’s test confirmed significantly greater variability in pyrosequencing measurements relative to TMS (*p* = 2.2 × 10^−16^), indicating increased dispersion of absolute methylation values. Analysis at the individual CpG level showed that all CpG sites exhibited higher methylation values when measured by pyrosequencing, although the magnitude of these differences varied substantially across loci (Figure 2(b)). Relative analyses based on percent differences with respect to TMS revealed consistent patterns (Suppl. Figure 2). The largest absolute difference was observed at CpG 44 (12.3%) (Suppl. Figure 3). Relative differences were greatest at CpGs 46 and 47, highlighting pronounced CpG-dependent effects on inter-method measurement behavior (Suppl. Figure 3).

**Figure 2.**
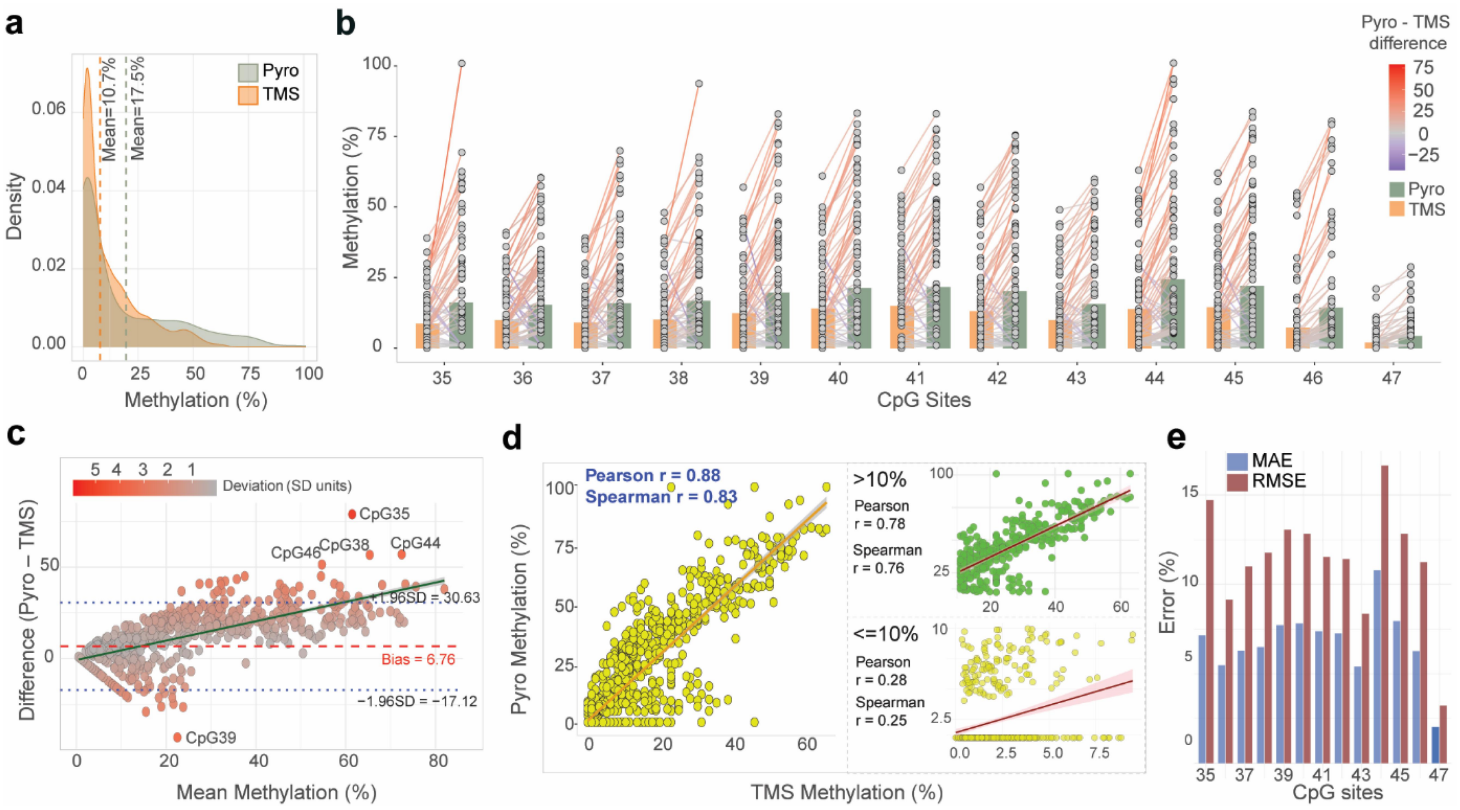
**Analysis of DNA methylation quantification between TMS and pyrosequencing**. (**a**) Density plots comparing global methylation distributions across all CpG sites for TMS and pyrosequencing methods. (**b**) Methylation levels per CpG site across both methods. Bar plots show the mean methylation per CpG and samples are overlayed as dots to the graph. (**c**) Bland-Altman plot assessing inter-methodology agreement. The mean difference and 95% limits of agreement are indicated. The top five outlier CpGs with large deviations are annotated. (**d**) Correlation coefficients per CpG, reflecting site-specific concordance between methods. Correlation coefficients between methods within low (≤10%) and moderate-to-high (>10%) methylation bins, indicating variable concordance by methylation level. (**e**) Root mean square error (RMSE) and mean absolute error (MAE) computed per CpG to quantify inter-methodology variability.

Inter-method agreement was evaluated using Bland-Altman analysis, which identified a mean difference of +6.76% with wide 95% limits of agreement (−17.12% to +30.63%), reflecting substantial variability in absolute methylation estimates across CpG sites (Figure 2(c)). Regression analysis further demonstrated a proportional bias, with larger positive differences observed at CpGs with higher average methylation levels and reduced or negative differences at CpGs with lower methylation levels, indicating that measurement discrepancies depend on both CpG site and methylation level. The slope of the Bland-Altman regression line shows that absolute method discrepancies increase as the mean methylation level rises, rather than reflecting a simple fixed additive offset.

Strong correlations were observed across all CpGs and samples, with Spearman’s rank correlation (ρ = 0.83) indicating preservation of relative ranking and Pearson’s correlation (*r* = 0.88) reflecting high linear association (Figure 2(d)). The difference between these correlation metrics is consistent with CpG-specific deviations captured by the Bland-Altman analysis. While correlation metrics capture preservation of relative rank order, Bland-Altman analysis assesses absolute agreement, two conceptually distinct properties that should not be conflated in interpretation. To assess the dependence of inter-method concordance on methylation level, samples were stratified into low (≤10%) and moderate-to-high (>10%) methylation bins. Correlation analyses revealed weak agreement in the low-methylation range (Pearson *r* = 0.29; Spearman ρ = 0.26) and substantially stronger concordance at higher methylation levels (Pearson *r* = 0.78; Spearman ρ = 0.76) (Figure 2(d)). Heteroscedasticity in inter-method differences was confirmed using the Breusch–Pagan test (*p* = 2.2 × 10^−16^), indicating that the variance of measurement differences depends on methylation level. CpG-specific error analysis using mean absolute error (MAE) and root mean square error (RMSE) highlighted the largest discrepancies at CpG 44, reinforcing the presence of site-specific measurement variability (Figure 2(e)). A ranked summary of MAE and RMSE per CpG is provided in Supplementary Table 5.

Overall, these results demonstrate that while pyrosequencing and TMS consistently preserve relative methylation trends at the *BACH2* promoter, absolute methylation values are not directly interchangeable and differ in a CpG- and methylation-dependent manner, with reduced agreement at low methylation levels.

## Context-dependent agreement and site-specific variability in DNA methylation quantification between TMS and EPIC array

To evaluate inter‑method variability between TMS and the EPIC, we compared DNA methylation measurements across 8 CpG sites shared between the two platforms in 90 samples spanning a range of methylation levels at the *BACH2* promoter (Figure 1(a)). Global distributions of methylation values obtained by EPIC and TMS showed similar central tendencies, with mean methylation levels of 9.5% and 9.2%, respectively (Figure 3(a)). Density plots indicated substantial overlap between the two methods, and Levene’s test revealed no significant difference in overall variance (*p* = 0.54), suggesting comparable dispersion of methylation values at the global level. At the individual CpG level, most sites showed highly comparable methylation estimates across methods; however, several CpGs showed reproducible deviations, indicating locus‑specific differences in measurement behavior (Figure 3(b)). Most CpGs showed minimal differences, whereas a subset of sites (CpGs 8, 46, 48, and 52) exhibited reproducible directional differences, with consistently higher methylation values reported by either EPIC or TMS across samples (Figure 3(b); Suppl. Figure 4). Importantly, the magnitude of these differences was modest and distinctly smaller than the discrepancies observed between pyrosequencing and TMS. Relative analyses based on percent differences with respect to TMS revealed consistent patterns (Suppl. Figure 4 & Suppl. Figure 5). Agreement between EPIC and TMS was further evaluated using Bland-Altman analysis, which revealed a mean difference of 0.37% with 95% limits of agreement spanning −17.37% to +18.11% (Figure 3(c)). Unlike the Pyro-TMS comparison (systematic bias = +6.76%), the EPIC–TMS discordance is characterised by near-zero mean bias (0.37%) with wide limits of agreement, indicating that disagreement reflects random site-level variability rather than a systematic directional offset, a distinction with important practical implications. While most data points fell within these limits, several CpGs (notably CpG 46, CpG 48, and CpG 52) exhibited larger deviations, highlighting the presence of site‑specific effects. Regression analysis further indicated a subtle proportional bias, with EPIC reporting slightly lower methylation values at higher methylation levels (Figure 3(c)).

**Figure 3.**
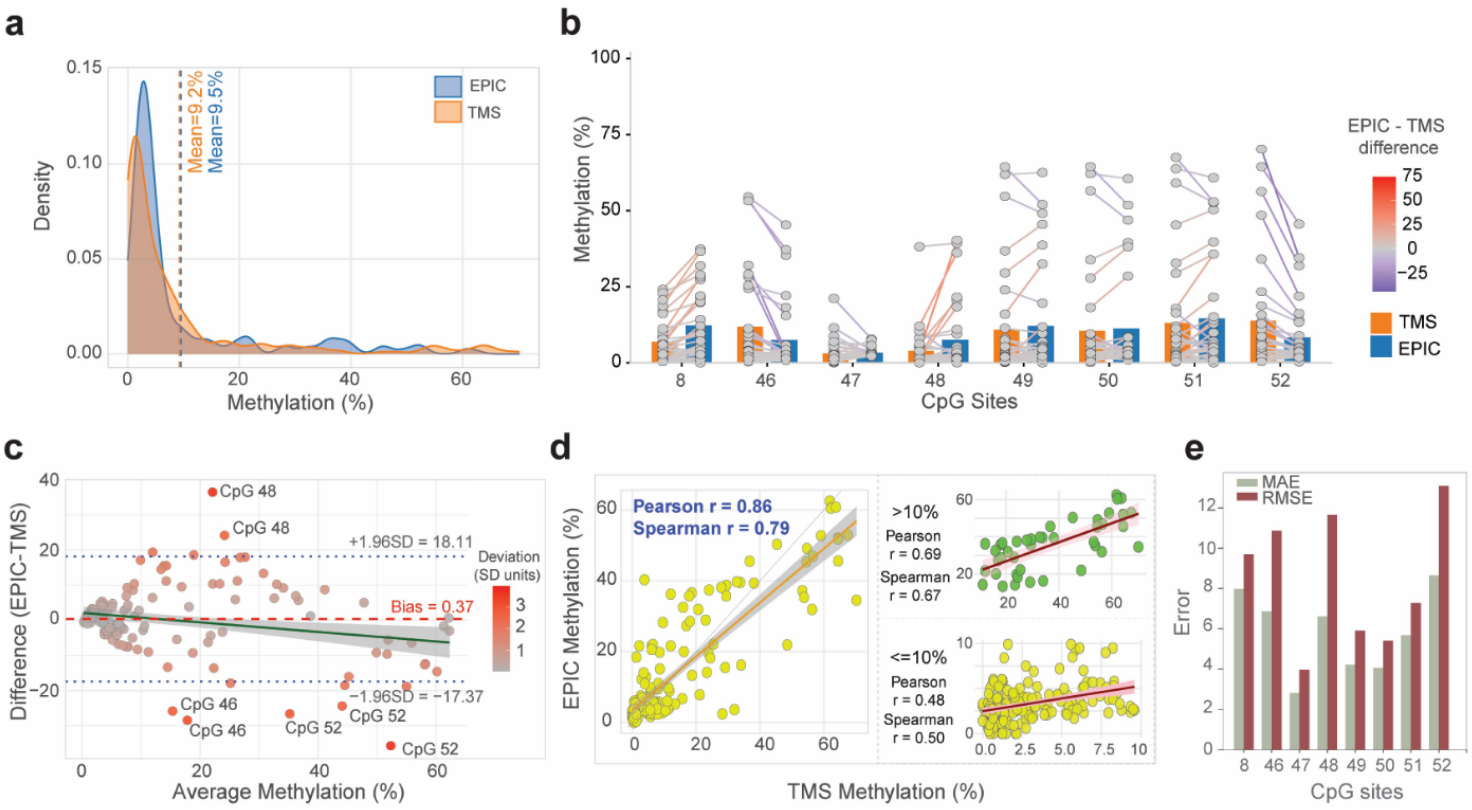
**Analysis of DNA methylation measurements between TMS and EPIC array. (a)** Density plot comparing the global methylation value distributions for TMS and EPIC methods. **(b)** Methylation levels per CpG site across both methods. Bar plots show the mean methylation per CpGs and samples are overlayed as dots to the graph. **(c)** Bland–Altman plot depicting agreement between methods across CpGs. The mean methylation difference (0.37%) and 95% limits of agreement (−17.37% to +18.11%) are shown. Outlier CpGs with large deviations are annotated. **(d)** Scatter plot showing overall correlation of methylation percentages between TMS and EPIC across matched CpG sites. Pearson correlation coefficients between methods within low (≤10%) and moderate-to-high (>10%) methylation bins, indicating variable concordance by methylation level. **(e)** RMSE and MAE are computed per CpG to quantify variability between methods.

Global correlation analysis demonstrated a strong linear association between methods (Pearson *r* = 0.86; Spearman ρ = 0.79) across all CpGs and samples (Figure 3(d)). To assess the influence of methylation level on inter‑method agreement, samples were stratified into low (≤10%) and moderate‑to‑high (>10%) methylation bins. Correlation analyses revealed diminished agreement in low-methylation range (Pearson *r* = 0.48; Spearman ρ = 0.5) and stronger concordance at higher methylation levels (Pearson *r* = 0.69; Spearman ρ = 0.67) (Figure 3(d)). Consistent with this observation, the Breusch–Pagan test indicated significant heteroscedasticity in inter‑method differences across methylation levels (*p* = 8.81 × 10^−13^), demonstrating increased variability at low methylation levels. CpG‑specific error analysis using MAE and RMSE showed that CpGs 8, 46, 48, and 52 exhibited the highest error values, whereas other sites maintained lower error metrics, reinforcing the presence of site‑ and methylation‑dependent variability (Figure 3(e)). A ranked summary of MAE and RMSE values per CpG is provided in Supplementary Table 6.

Collectively, these results indicate that while EPIC and TMS exhibit overall consistency in relative DNA methylation patterns at the *BACH2* promoter, absolute methylation estimates are influenced by both CpG‑specific and methylation‑dependent effects. Reduced agreement and increased variability at low methylation levels underscore the need for cautious interpretation of EPIC‑derived absolute values in targeted, low‑methylation contexts.

## Illustrative cross-platform comparison at CpG sites shared across EPIC, TMS, and pyrosequencing

Two CpG sites (CpGs 44 and 43) were shared across all three platforms enabling direct three‑way comparison in 90 samples spanning a range of methylation levels at the *BACH2* promoter (Figure 1(a); Suppl. Table 2). Given the limited number of shared loci, these analyses are intended to illustrate CpG‑ and methylation‑dependent measurement behavior rather than to support platform‑wide generalizations. Across both CpGs, density plots revealed method‑specific differences in methylation distributions (Figure 4(a)). The EPIC array exhibited broader variability, particularly at lower methylation levels, whereas TMS and pyrosequencing showed more compact distributions, with pyrosequencing slightly shifted toward higher methylation values. Principal component analysis (PCA) indicated that the first principal component (PC1; 73% variance) was primarily driven by overall methylation level rather than measurement platform, with samples clustering according to mean methylation rather than assay type (Figure 4(b), Suppl. Fig 6). Samples with the highest mean methylation clustered distinctly along PC1, further confirming that separation in PCA space reflects methylation state rather than platform-specific effects. We acknowledge that the PCA was performed on a limited number of observations due to low overlap between CpGs across the measurement platforms, requiring careful interpretation of the results.

**Figure 4.**
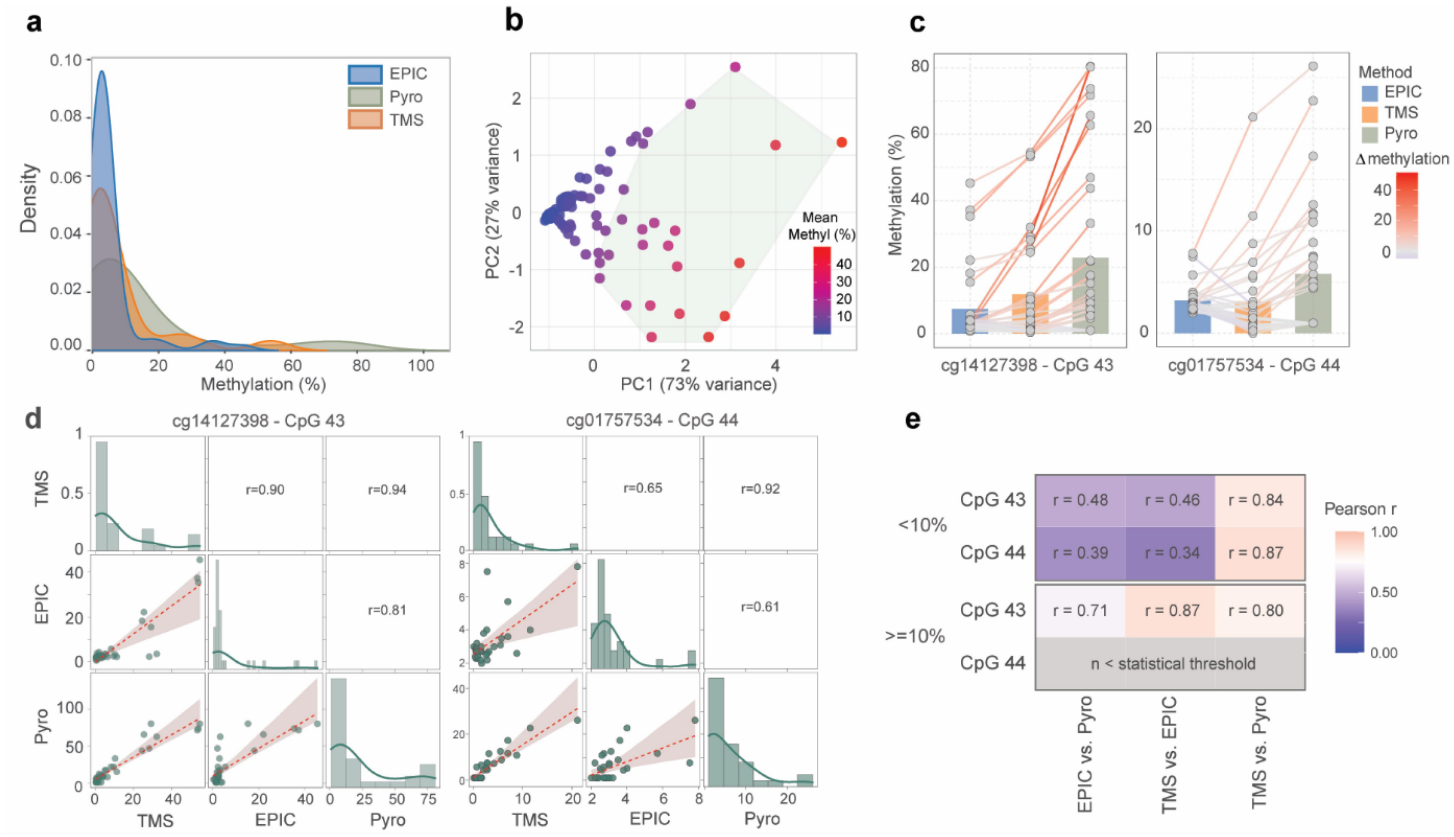
**Comparative analysis of DNA methylation measurements across three methods for two shared CpG sites. (a)** Density plots showing the distribution of methylation levels across all samples for each method. EPIC exhibited broader variability, particularly at low methylation levels. **(b)** Principal component analysis (PCA) of methylation profiles across EPIC, TMS, and pyrosequencing platforms. Each point represents a sample-method combination, positioned by the first two principal components derived from CpG methylation values. Point fill indicates the mean methylation level (blue = lower, red = higher. The shaded polygon highlights samples in the top 25% of mean methylation (high-methylation group), illustrating clustering patterns in PCA space. **(c)** Per-CpG comparison of DNA methylation measurements across platforms. for each CpG site, bars represent the mean methylation level, while individual points denote sample-level measurements. Line segments connect paired measurements from the same sample across adjacent platforms, with segment color indicating the direction and magnitude of methylation change (Δ methylation). **(d)** Pearson correlation coefficients between methods for each CpG site. **(e)** Pairwise correlation plots for each CpG site, stratified by methylation level (<10% and ≥10% based on TMS). Correlations were lower in the <10% bin, especially for EPIC vs pyrosequencing, and improved markedly in the ≥10% bin.

Per‑CpG comparison of paired measurements demonstrated that inter‑method differences occurred in a CpG‑specific manner (Figure 4(c)). CpG 44 was characterized by consistently low methylation across samples and exhibited greater variability and reduced agreement in EPIC‑involving comparisons, whereas CpG 43, which showed higher methylation levels, displayed more consistent measurements across platforms. Statistical testing using the Friedman test confirmed significant differences among platforms for both CpG 43 (χ^2^(2) = 22.24, *p* < 0.0001; Kendall’s W = 0.34) and CpG 44 (χ^2^(2) = 14.36, *p* = 0.0008; W = 0.22), indicating moderate concordance with systematic platform‑dependent differences. Reliability analysis using the intraclass correlation coefficient (ICC) further underscored CpG‑specific behavior. CpG 44 showed moderate agreement across platforms (ICC = 0.551; *p* = 0.0008), whereas CpG 43 exhibited higher concordance (ICC = 0.748; *p* < 0.0001), consistent with improved agreement at higher methylation levels (Suppl. Table 7). Pairwise correlation analyses similarly demonstrated stronger concordance across methods for CpG 43 compared with CpG 44 (Figure 4(d)). To assess the influence of methylation level on inter‑platform agreement, samples were stratified into low (<10%) and moderate‑to‑high (≥10%) methylation bins (Figure 4(e)). At low methylation levels, CpG 43 showed the highest correlation between TMS and pyrosequencing (*r* = 0.84), with reduced correlations involving EPIC (TMS-EPIC *r* = 0.46; EPIC-pyrosequencing *r* = 0.48). Similar patterns were observed for CpG 44, where EPIC‑involving comparisons showed diminished concordance at low methylation levels. At higher methylation levels, correlations improved across all method comparisons, particularly for CpG 43.

Together, these analyses illustrate that inter‑platform agreement is strongly dependent on both CpG site and methylation level. While relative methylation trends are preserved across platforms, absolute methylation values and inter-platform agreement diverge most prominently at low methylation levels, particularly for EPIC‑involving comparisons. Given the limited number of shared CpG sites, these findings should be interpreted as illustrative of context‑dependent measurement behavior rather than as evidence of generalizable platform performance.

## Discussion

Accurate and reproducible quantification of DNA methylation at single‑CpG resolution is essential for both functional epigenetic studies and translational applications. In this study, we performed a controlled, locus‑specific comparison of three widely used bisulfite‑based DNA methylation assays, pyrosequencing, targeted methyl sequencing (TMS), and the Infinium MethylationEPIC array, using identical DNA samples generated by CRISPR/dCas9‑mediated epigenome editing at the BACH2 promoter. By decoupling technical variability from biological heterogeneity, our analyses reveal that while relative methylation trends are consistently preserved across platforms, absolute methylation values are method‑, CpG‑, and methylation‑dependent, with reduced agreement at low methylation levels.

Across all method comparisons, methylation level emerged as a dominant factor influencing inter-method agreement. Both TMS-pyrosequencing and TMS-EPIC comparisons showed substantially reduced concordance and increased variability below approximately 10% methylation, whereas agreement improved markedly at moderate-to-high methylation levels. This behavior was reflected consistently across Bland-Altman analyses, correlation stratification, and error-based metrics (MAE and RMSE), indicating that correlation alone is insufficient to characterize quantitative agreement between platforms, particularly in low-methylation contexts. CpG-specific effects further contributed to inter-method variability. In the comparison between TMS and pyrosequencing, CpGs located at greater distances from the sequencing primer (e.g., CpGs 35–40) exhibited reduced concordance, consistent with position-dependent signal deterioration and PCR-associated amplification bias reported for bisulfite-based assays [24–26]. These effects are well documented and arise from cumulative signal decay, incomplete extension, and differential amplification efficiency along the sequencing read [27,28]. Similarly, in EPIC-TMS comparisons, reproducible deviations at specific CpGs (e.g., CpGs 8, 46, 48, and 52) likely reflect probe-specific hybridization behavior influenced by local sequence composition and GC content rather than random measurement noise as previously documented for Illumina EPIC array measurements [29,30]. Notably, Type II probes on the EPIC array exhibit compressed dynamic range relative to Type I probes, which may disproportionately affect quantitative accuracy at low methylation levels [29].

The proportional nature of this bias (discrepancies increasing with methylation level, as visible from the Bland-Altman regression slope in Figure 2c) may reflect differential amplification efficiency of methylated versus unmethylated bisulfite-converted templates as a function of local CpG density [24,26]. Reduced agreement at low methylation levels may also be influenced by stochastic sampling effects associated with limited sequencing depth. We directly assessed this possibility: TMS read depth at this panel was high overall (median 1,720×; minimum 18×) and supported sub-percent resolution even among the lowest-covered CpGs and at low methylation levels (Suppl. Figure 1), indicating that limited sequencing depth is unlikely to be a major driver of the reduced agreement observed below 10% methylation in our study. Although a minimum coverage threshold of 10× was applied for TMS, CpGs with low absolute methylation remain inherently more susceptible to sampling variance, which can inflate apparent inter-method differences. Future analyses incorporating systematic subsampling or read-depth-stratified modeling would be valuable for quantitatively assessing the contribution of coverage depth to measurement precision in this regime. A limitation of this study is the use of different bisulfite conversion kits across platforms. Pyrosequencing and EPIC array measurements relied on the EZ DNA Methylation-Gold kit, whereas TMS libraries were generated using the EpiTect Fast Bisulfite Conversion kit. Differences in conversion efficiency, DNA degradation rates, and fragment recovery between chemistries may therefore contribute to systematic offsets in absolute methylation values. However, an independent, controlled comparison of nine bisulfite conversion kits, including both kits used in this study, found conversion efficiencies within a narrow range (98.7–99.9%) that were considered unlikely to meaningfully impair downstream analysis [31]. While identical DNA samples were processed across platforms to minimize biological variability, future studies applying standardized bisulfite conversion workflows will be required to fully disentangle biochemical from platform-specific sources of variation. Consistent with these technical constraints, multiple comparative studies have reported that bisulfite pyrosequencing tends to report higher absolute methylation values than alternative locus-targeted methods, particularly in low-methylation contexts and clinical samples [27,28,32].

Three-way comparison across EPIC, TMS, and pyrosequencing was necessarily limited to two CpG sites shared across all platforms. Analyses at these loci demonstrated that inter-platform agreement remained strongly CpG- and methylation-dependent, with improved concordance at higher methylation levels and reduced agreement, particularly for EPIC-involving comparisons, at low methylation. Given the extremely limited number of shared CpGs, these two-site comparisons should be treated as illustrative examples of the CpG- and methylation-dependent patterns described above, rather than as a basis for generalizable claims about platform-wide performance. EPIC array performance has also been shown to vary as a function of probe design and genomic context across diverse datasets and populations, further emphasizing the importance of CpG-aware interpretation of array-based methylation measurements [30,32].

Additional limitations should be noted. First, the analysis was restricted to a single promoter region (*BACH2*), and measurement behavior may differ in genomic regions with distinct CpG density, sequence context, or chromatin accessibility. Future studies should validate these findings at additional loci. Second, EPIC array data were available for a subset of samples, resulting in unequal sample sizes across platforms that may modestly influence correlation and error estimates. Finally, this study focused exclusively on bisulfite-based chemistries; enzymatic and nanopore-based approaches may exhibit distinct bias profiles and warrant separate systematic evaluation. Preliminary evidence from independent comparisons suggests that both EM-seq [33] and Oxford Nanopore sequencing [34] can achieve broadly comparable DNA methylation estimates relative to bisulfite- and array-based methods, though full concordance was not observed in either comparison. This indicates that inter-platform agreement is methylation-level-dependent across distinct measurement technologies, even though the specific range of divergence differs between studies.

From a practical standpoint, these findings emphasize that method selection should be driven by experimental objectives and expected methylation ranges rather than assumptions of cross-platform interchangeability. The EPIC array remains well suited for large-scale discovery studies, TMS enables scalable and high-resolution targeted profiling, and pyrosequencing remains valuable for rapid, low-throughput locus validation. However, when precise absolute methylation quantification is required, particularly at low methylation levels, method-aware interpretation and orthogonal validation are essential. In particular, EM-seq, which uses enzymatic rather than chemical bisulfite conversion, offers improved conversion efficiency and reduced DNA fragmentation. Assessing whether the systematic positive bias seen for pyrosequencing and the low-methylation variability of the EPIC array are reduced or absent with EM-seq would be a valuable prospective direction. In addition, the predominantly low mean methylation observed across the sampled CpGs and conditions likely reflects both the range of editing states captured by our experimental design and the intrinsic hypomethylation typical of active gene promoters. As a result, the CpGs and methylation range sampled here are not representative of the bimodal, genome-wide distribution of DNA methylation, and conclusions, particularly the two-CpG three-way comparison, should be interpreted accordingly.

## Conclusion

In summary, this locus‑specific comparison demonstrates that bisulfite‑based DNA methylation assays consistently capture relative methylation patterns but differ systematically in absolute values as a function of CpG context and methylation level. Rather than identifying a single optimal platform, this work delineates measurable contexts in which inter‑method agreement is robust and others in which caution is warranted, providing a practical framework for interpreting and integrating locus‑specific DNA methylation data.

## Supplementary Material

Rangani_et al_Supplementary Figures_R1.docx

Rangani et al_Supl_Tables_Final_R1.xlsx

## Data Availability

All data related to the study is presented in the article and in the supplementary material. Per-sample, per-CpG methylation measurements for all three platforms (EPIC array, TMS, and pyrosequencing) are provided in Supplementary Tables 3 & 4.
