# Supplementary material for: CpG site- and methylation level-dependent measurement variability across three bisulfite-based DNA methylation assays
Source: Epigenetics. 2026 Oct 29;21(1):2753261. doi: 10.1080/15592294.2026.2753261 (PMC13733195; doi:10.1080/15592294.2026.2753261)
Supplement: Supplementary file 1 [file KEPI_A_2753261_SM6673.docx]

**Supplementary Figure 1. TMS read depth and measurement resolution at the *BACH2* promoter panel.** Read depth per CpG per sample and methylation values for all 86 CpG sites and 90 samples profiled by TMS at the *BACH2* promoter (7,740 CpG × sample measurements in total). **(a)** Distribution of read depth across all measurements (log scale). The dashed red line indicates the median depth (1,720X); the dotted grey line indicates the 10X coverage threshold applied for CpG exclusion (see Methods). **(b)** Median read depth (blue line/red points) and full min-max range (shaded band) per CpG site, plotted by position along the *BACH2* amplicon (bp from the first covered CpG). The amber band (chr6:90,296,952-90,297,083) marks the primer-covered region for which the primer design tool predicted lower performance, which coincides with the observed depth trough. **(c)** Achievable measurement resolution (log scale), defined as the smallest detectable change in methylation (%) given the read depth at each CpG-sample measurement (100 ÷ read depth), plotted against the corresponding TMS methylation level (%) for each of the 7,740 measurements. The dashed red line marks the 10% methylation threshold used to define the low- and moderate-to-high methylation bins in stratified analyses (Figs. 2d, 3d, 4e). The dotted grey line marks 1 percentage point, the resolution at which TMS methylation values are reported (nearest whole percent) in the manuscript and source data, shown here for comparison against the depth-based resolution achievable at each point.

**Supplementary Figure 2.** **Comparison of CpG methylation measured by TMS and Pyrosequencing (Pyro) across samples.** Bars represent the mean methylation (%) per CpG site for each method (orange = TMS, green = Pyro). Individual sample measurements are shown as gray circles, and lines connect the same sample across methods for each CpG site. **(a)** The plot shows the absolute difference (Pyro – TMS) per sample, highlighting the magnitude of change. **(b)** The plot shows the percent increase relative to TMS per sample, illustrating relative changes normalized to the initial methylation level. Line colors indicate the magnitude of change (blue = negative, gray = no change, red = positive).

**Supplementary Figure 3.** **Comparison of methylation bias between targeted methylation sequencing (TMS) and pyrosequencing across BACH2 CpG sites.** **(a)** Absolute bias, calculated as the difference in mean methylation detected by pyrosequencing minus TMS for each CpG site. **(b)** Relative bias, expressed as the percent increase of pyrosequencing values relative to TMS. Positive values indicate higher methylation detected by pyrosequencing, while negative values indicate higher methylation detected by TMS.

**Supplementary Figure 4.** **Comparison of CpG methylation measured by TMS and EPIC arrays across samples.** Bars represent the mean methylation (%) per CpG site for each method (orange = TMS, blue = EPIC). Individual sample measurements are shown as gray circles, and lines connect the same sample across methods for each CpG site. **(a)** The plot shows the absolute difference (EPIC – TMS) per sample, highlighting the magnitude of change. **(b)** The plot shows the percent increase relative to TMS per sample, illustrating relative changes normalized to the initial methylation level. Line colors indicate the magnitude of change (blue = negative, gray = no change, red = positive).

**Supplementary Figure 5.** **Comparison of methylation bias between targeted methylation sequencing (TMS) and EPIC array across BACH2 CpG sites.** **(a)** Absolute bias, calculated as the difference in mean methylation detected by EPIC minus TMS for each CpG site. **(b)** Relative bias, expressed as the percent increase of EPIC values relative to TMS. Positive values indicate higher methylation detected by EPIC, while negative values indicate higher methylation detected by TMS.

**Supplementary Figure 6.** **Principal Component Analysis (PCA) of methylation profiles across EPIC, TMS, and pyrosequencing platforms.** Each point represents a sample-method combination, positioned by the first two principal components derived from CpG methylation values. Point fill indicates the mean methylation level (blue = lower, red = higher), and the measurement method is shown as a colored ring around the point: EPIC (blue), TMS (orange), and pyrosequencing (green).
